# Comments on “Learning needs and experiences of hospital nurses in genetic medicine in a rural area of Japan: A cross-sectional questionnaire survey in Oita prefecture”

**DOI:** 10.1016/j.pmedr.2026.103399

**Published:** 2026-01-28

**Authors:** Hawkar A. Nasralla, Hussein Mustafa Hamasalih, Fahmi H. Kakamad

**Affiliations:** a Scientific Affairs Department, Smart Health Tower, Madam Mitterrand Street, Sulaymaniyah, Iraq; b Bright Technical and Vocational Institute, Department of Nursing, Sulaymaniyah, Iraq; c College of Medicine, University of Sulaimani, Madam Mitterrand Street, Sulaymaniyah, Iraq; d Kscien Organization for Scientific Research (Middle East Office), Sulaymaniyah, Iraq

To the editor

We read with great interest the article by Tsukatani et al., which provides valuable insights into nurses' perceptions and educational needs regarding genetic medicine in Oita Prefecture, a representative rural region of Japan (Tsukatani et al., 2025). While we commend the authors for addressing this underexplored topic, and acknowledge that they appropriately recognized several important limitations of their study, we believe that additional methodological considerations warrant further discussion, as they may influence the interpretation of the findings.

First, the potential for sampling and non-response bias. The valid response rate was approximately 53% (1001/1881). Yet, the authors did not analyze differences between respondents and non-respondents (e.g., age, experience, interest in genetics) or discuss how non-response might bias the results. Prior research among healthcare workers shows that non-respondents may differ systematically in demographic and attitudinal variables (e.g., younger staff, less affiliated, less experienced), which can distort survey findings (Listyowardojo et al., 2011). Without such assessment, the sample may over-represent nurses more interested in genetics or education, leading to inflated estimates of “learning needs” or awareness.

Second, Although the authors appropriately limit their conclusions to Oita Prefecture, some statements in the discussion may nonetheless be interpreted as having broader relevance to rural nursing contexts in Japan. Given that the sample was restricted to cancer treatment hospitals within a single prefecture, further emphasis on the contextual specificity of these findings would help prevent overextension of their applicability. Replication across diverse rural and non-cancer hospital settings would be necessary before broader inferences can be made. The literature on nursing research underscores that findings from specific contexts may not translate directly to community hospitals or different geographic settings (Sharp, 1998).

Third, there are concerns about measurement validity and the exclusive reliance on self-reported data. The questionnaire employed in the study had not been validated. Strong content validity is fundamental when developing or selecting an outcome measurement tool; without it, findings may be misleading and lead to incorrect conclusions (Mokkink et al., 2025). Moreover, the study depends solely on participants' self-assessed understanding of genetics and their self-identified learning needs. However, evidence shows that self-ratings often correlate poorly with objective knowledge. For instance, a multi-site study reported minimal correlations (*r* = 0.017–0.123) between nurses' self-reported evidence-based practice knowledge and objective assessment scores (Hagedorn Wonder et al., 2017). Therefore, participants perceived “understanding” may not accurately represent their actual knowledge or competence in genetic medicine. In the absence of objective measures (e.g., knowledge tests or case-based evaluations), interpretation of “understanding” is limited and may mischaracterize the true educational gaps.

Finally, contextual and procedural factors may also have influenced responses. The survey period coincided with the COVID-19 pandemic, which substantially affected nurse workloads, stress levels, and access to continuing education opportunities. In addition, distribution of questionnaires via nursing directors, despite anonymity, may have introduced social desirability or hierarchical response bias. These factors could have shaped participants' self-reported perceptions and warrant consideration when interpreting the findings (Van de Mortel, 2008; Roustaei et al., 2015).

Future studies could address these limitations by assessing potential non-response bias through demographic comparisons or sensitivity analyses, expanding sampling beyond cancer hospitals to improve generalizability, and incorporating validated instruments or objective knowledge assessments alongside self-reported measures. Consideration of contextual factors such as pandemic-related workload and the use of independent survey distribution methods may further strengthen methodological rigor and reduce response bias.

## CRediT authorship contribution statement

**Hawkar A. Nasralla:** Writing – review & editing, Writing – original draft, Conceptualization. **Hussein Mustafa Hamasalih:** Writing – review & editing. **Fahmi H. Kakamad:** Writing – review & editing, Validation, Supervision.

## Funding/Support

No funding was received for this work.

## Declaration of competing interest

The authors declare that they have no known competing financial interests or personal relationships that could have appeared to influence the work reported in this paper.

## Data Availability

No data was used for the research described in the article.
